# Pulmonary Hypertension and Hypothyroidism—Still an Important Clinical Coincidence in Paediatric Population, an Endocrinologist’s Point of View

**DOI:** 10.3390/life14030302

**Published:** 2024-02-26

**Authors:** Agnieszka Lecka-Ambroziak, Karolina Kot

**Affiliations:** 1 Endocrinology Outpatient Clinic, Institute of Mother and Child, 01-211 Warsaw, Poland; 2 Department of Endocrinology and Diabetology, The Children’s Memorial Health Institute, 04-730 Warsaw, Poland; k.kot@ipczd.pl

**Keywords:** pulmonary hypertension, pulmonary arterial hypertension, autoimmune thyroid disease, hypothyroidism, growth impairment, genetic disorders, registries

## Abstract

There is limited data on hypotheses linking autoimmune thyroid diseases (AITD) and hypothyroidism with pulmonary hypertension (PH). Moreover, the prevalence of this coincidence, as well as the possible common pathogenic mechanisms, are even less explicit in paediatric population. We present a review of recently published articles regarding relatively large cohorts of children with PH, coming from paediatric PH registries, aiming to clarify the coincidence of PH and AITD, especially hypothyroidism, and discuss its possible mutual impact. Although thyroid disorders have been excluded from the latest PH classification, it is still important to remember the possibility of this coincidence as it may significantly influence patients’ clinical outcome. Moreover, children with PH may need multidisciplinary care due to the relatively frequent coexistence with not only hormonal abnormalities but also growth impairment, genetic disorders, and mental delay. Further specific paediatric studies are needed to improve the care in this rare disease, especially in patients with other comorbidities present.

## 1. Introduction

Pulmonary hypertension (PH) is a chronic and often progressive disease that not uncommonly may be caused or influenced by other comorbidities [1]. The prevalence of autoimmune thyroid diseases (AITDs) in patients with PH has been estimated as high as 24% [1,2,3,4] and even higher in studies regarding smaller cohorts of patients [5,6]. Pulmonary arterial hypertension (PAH), with an estimated incidence of 4–10 cases per million children per year [7,8], is described as a PH subgroup with the highest prevalence of AITDs [1]. On the other side, the risk of PH in populations with AITDs and hypothyroidism is not well studied and there are mainly case reports published [7,9]. PH is categorized into five main clinical groups, with further differentiation into specific subgroups [8,10,11].

When analysing the influence of thyroid diseases on PH, it is worth remembering that thyroid hormones act directly on the cardiovascular system through thyroid hormone receptors located in the myocardial and vascular endothelial tissues. Other indirect actions depend on the adrenergic nervous system or renin–angiotensin–aldosterone system [1,12]. Both hypothyroidism and hyperthyroidism may lead to PH [1,13]. It is hypothesised that AITD and PH may have similar pathogenic backgrounds, from autoimmunity, to heart dysfunction, to angioproliferation profiles [1,10]. However, it was recently decided at the Sixth World Symposium on PH in 2018 to withdraw thyroid disorders from the PH classification (PH Group 5) [10]. Nevertheless, it is still underlined that AITDs may play significant role as risk factors and/or associated comorbidities [10,14]. Many authors underline the necessity of performing the thyroid function tests in patients newly diagnosed with PH, as well as regularly monitoring the thyroid function thereafter [12,14]. It seems to be even more important in paediatric populations, as coincidence of these diseases may significantly influence growth and development of a child [5,7,15].

This article aims to present a review of recently published articles on the coincidence of PH and AITD, especially hypothyroidism, in paediatric populations and discusses its possible impact on patients’ clinical outcome. This is a narrative review and mainly regards the paediatric PH/PAH registries.

### Review of the Literature

We reviewed the recent publications, focusing mainly on paediatric PH registries. The articles were published during the last 15 years and represent data from the last 30 years. They include national as well as international registries and both prospective and retrospective analyses. It is important to mention that although the studies were conducted in Europe and the United States, the results may differ between the countries and have to be interpreted with caution. The PH prevalence, natural history, and outcome may depend on such factors as lifestyle and environmental issues, degree of access to modern diagnostic tests and treatment procedures, differences in management standards, and general awareness of the disease among the population and the healthcare providers [7]. We summarise the review in Table 1 and Table 2.

In a prospective study from 2020 presenting the results from the paediatric arm of the Polish Registry of Pulmonary Hypertension, hypothyroidism was one of the most frequent comorbidities among 80 prospectively enrolled children with PAH in 2018 [7]. Hypothyroidism incidence was 23.8% and was the third comorbidity after decreased growth velocity and mental delay. Hypothyroidism was more common in PAH associated with congenital heart disease (CHD-PAH), in 16 out of 54 patients, than in patients with idiopathic PAH (IPAH), in 3 out of 25 patients. It is important to mention that hypothyroidism was frequently diagnosed in children with Down syndrome, in 15 out of 22 patients with CHD-PAH and in 1 of 2 patients with IPAH. Authors comment that the presented prevalence of hypothyroidism in the Polish paediatric PAH population is much higher than in the population of children with PAH in the United States (US), where thyroid disorders were present in 2.7% [23]. Another analysis from the US of 1475 children from the Pediatric Pulmonary Hypertension Network (PPHNet) showed data regarding thyroid replacement therapy in 4% [24]. This discrepancy may result from the large proportion of children with Down syndrome, with 24% of the Polish study group, in comparison to the analyses in American populations, with 12.5% and 10.7%, respectively. However, even in this subgroup of patients, hypothyroidism was more frequent than previously reported.

The incidence of hypothyroidism, as well as diagnosis of genetic syndromes, in children with PH vary significantly between the studies. In the Spanish registry among 142 patients with PH, hypothyroidism was present in 14.3%, and children with Down syndrome represented 17% of the study group [22]. Two publications from The Netherlands showed similar proportion of patients with Down syndrome: 18% of 154 children with progressive PAH and 21% of 63 patients with PH [16,21]. A United Kingdom (UK) study presenting a retrospective analysis of 64 children with IPAH showed that genetic syndromes were not as common; three patients had Down’s syndrome, one child had Noonan’s syndrome, and one had triple X syndrome [20]. However, the recent analysis of the whole cohort of patients with PH over twenty years from the same centre showed that genetic abnormalities were present in 34% of children, and Down syndrome was the most common (16%) [25]. Among the other genetic syndromes, the following were recognized in children with PH in different registries: Noonan, velocardiofacial, Di George, Patau, Edward, Cri-du-chat, Klinefelter, Jacobsen, fragile X, Prader–Willi, Silver–Russell, Marfan, Ehlers–Danlos, and Alagille [7,16,21,22,23,24]. Although these genetic diagnoses are not as common as Down syndrome in children with PH, it is important to remember that some of them are connected with higher frequency of thyroid diseases.

In a paper from 2010 assessing a small group of 16 patients diagnosed with IPAH below the age of 15 years, almost half of the group (44%) had AITD. In this subgroup, two patients developed Graves’ disease, two had silent thyroiditis, and in three, antithyroid antibodies were present with euthyroidism [5]. On the other hand, in an analysis of TOPP study (The Tracking Outcomes and Practice in Pediatric Pulmonary Hypertension) in the population of 362 patients with PH from 31 centres in 19 countries, between 2008 and 2010, only one patient with PAH was reported to have thyroid disease [18].

Analysis of the paediatric registries has its limitations, as the authors do not always present the data regarding coincidence with thyroid disorders or genetic anomalies [17,19]. Moreover, the registries vary in case of dedicated populations, either PH or only PAH/IPAH.

## 2. Discussion

**Figure 1:**
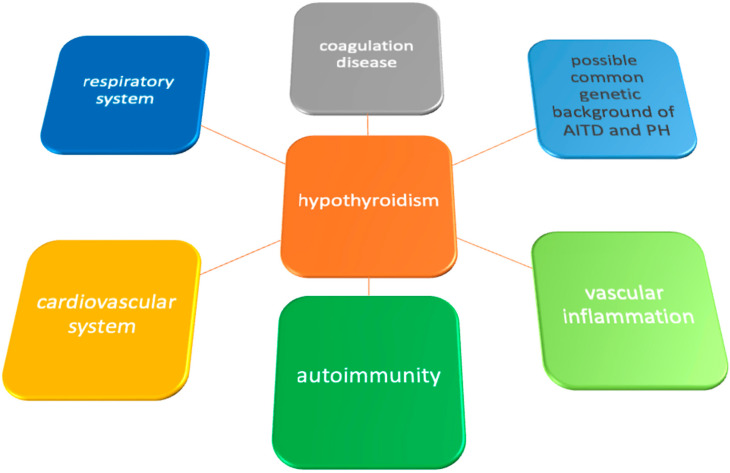
Presentation of possible links of hypothyroidism and pathogenetic factors in pulmonary hypertension.

It has been postulated that hypothyroidism in a course of AITD and PH, especially IPAH, may present common pathogenic mechanisms (Figure 1) [1].

Hypothyroidism has a direct effect on the cardiovascular system, with decreased cardiac contractility, increased diastolic blood pressure, and systemic vascular resistance [1,10,13,26,27,28]. On a molecular level, it decreases nitric oxide synthase expression and influences adenosine production, leading to decreased vasodilation. On the other hand, hypoventilation and hypoxia caused by hypothyroidism may also induce vasoconstriction. Furthermore, autoimmunity is hypothesised to play an important role in both diseases and may promote angioproliferation, vascular remodelling, and vascular inflammation [1,2]. Hypothyroidism may be also linked to coagulation disorders and increased thrombosis process [1].

Moreover, there is a hypothesis of a possible common genetic background of the diseases, as thyroid anomalies seem to be associated with *BMPR2* mutations (bone morphogenetic protein receptor type II gene), a well described genetic cause of PAH [1,24,29,30,31]. In a report regarding analysis of *BMPR2* mutations in both adult and paediatric patients with IPAH, there were five novel mutations found in 4 of 66 adults (6%) and in 1 of 75 children (1%) [29]. Thyroid disease was diagnosed in all five patients, compared to 24% of the adults and 5% of the children without mutations. Thyroiditis was present in three adults; one had follicular hyperplasia. A high titre of antimicrosomal antibodies was detected in the child and in one adult with thyroiditis. Although antinuclear antibodies were present in all adults with *BMPR2* mutations, autoantibodies characteristic of a specific connective tissue disease were absent [29]. The recent studies show higher frequency of *BMPR2* mutations in children with PH, but there is a lack of the analysis of possible coincidence with thyroid abnormalities. In the study from PPHNet Registry (US), *BMPR2* gene mutation was the most frequent cause in the group with heritable cases of PAH: 17 out of 40 children (43%) [24]. Clinical implications of the genetic background in paediatric PAH have become a new territory for future improvement of care of the patients [30]. The study in the Spanish PAH paediatric population REHIPED (REgistro de pacientes con HIpertensión Pulmonar PEDiátrica; Spanish Registry for Pediatric Pulmonary Hypertension) confirmed *BMPR2* as the most common cause of heritable PAH (10 cases, 64.7% of the group of patients with hereditary PAH after genetic studies). Among the whole group of 98 children enrolled in the years 2011–2021, in 44.9%, pathogenic or likely pathogenic different genetic variants were found, which led to a change of classification in 28.6% of the cohort. This reclassification had relevant implications regarding clinical prognosis. One of the conclusions of this publication is the necessity to widen the genetic background in future classifications of paediatric PH [30]. The difficulties in diagnosis of one specific PH group is consistent with a UK study from 2022 where among 1101 children with PH, multiple contributory causes were common and 16.9% of the cohort were displaying features of more than one diagnostic PH group [25].

Despite a strong rationale for the concomitance of thyroid diseases and PH, there was a consensus at the Sixth World Symposium on PH in 2018 to withdraw thyroid disorders from the classification as they do not characterise a specific clinical condition [10]. However, it was discussed that it is an important issue that needs to be controlled during the course of PH [8,10,12,14]. The data from the reviewed paediatric PH registries regarding thyroid diseases are scarce, as most of them do not present its exact frequency, and often there is lack of further detailed analysis of the specific thyroid disease diagnosis.

AITDs may influence the PH treatment outcome, and thyroid function evaluation is important to prevent further deterioration of right heart failure [5]. Furthermore, it is important to remember that childhood is characterised by rapid growth and development. As shown by paediatric PH registries, impaired growth may be a significant symptom in these patients [1,7,15], and decreased growth velocity may be aggravated by hypothyroidism. A UK retrospective study showed, through multivariable analysis, a very important association of low weight z-score with increased mortality in children treated for IPAH [20]. In another national analysis, a French prospective study of 50 patients, again, there was a significant proportion of children with PAH with low weight and height: 24% and 16% respectively [17]. Growth restriction was also reported by the authors of the recent UK analysis, with median z-score values for the whole large PH cohort: −1.1 (−2.1 to 0.1) for height and −1.4 (−2.7 to 0.3) for weight [25]. In an interesting analysis of four contemporary prospective registries of paediatric PAH, TOPP, REVEAL (The Registry to Evaluate Early and Long-Term PAH Disease Management), Dutch (Network for Diagnosis and Treatment of Pediatric PAH), and French national registries, representing 53 centres in 19 countries, 601 children were followed up for a median of 2.9 years [15]. To assess growth in children with PAH, the authors used the WHO 2006 growth standards of height for age and body mass index (BMI) for age. Both parameters expressed in z-scores were significantly lower than the reference, −0.81 (−0.93 to −0.69) and −0.12 (−0.25 to −0.01), respectively. Disturbed growth was particularly seen in younger children aged ≤5 years with IPAH or hereditary PAH and in all patients with CHD-PAH. The degree of growth impairment was independently associated with cause of PAH and comorbidities. As predictable, a favourable clinical course of PAH was associated with catch-up growth. It leads to a clinically useful conclusion that height for age could serve as an additional and easy-to-implement parameter to monitor the treatment outcome [15]. It has been postulated that there is a need for cooperation between PH physicians, endocrinologists, and dietary specialists while treating paediatric patients with PH [7].

What is necessary to discuss is the quality of life (QoL) in children with PH. French investigators in a paper cited above analysed the impact of PAH on scholarship and QoL in a subgroup of 40 patients over three years of age [17]. Most of the children, 81%, were attending regular school, of which three patients had learning disabilities and seven received tuition. For assessment of QoL, the authors used a Child Health Questionnaire—Parent Form 50 (CHQ-PF50), for children over five years old, at inclusion and after one and two years, comparing the results with the standard and asthmatic paediatric reference cohorts. The majority of children with PAH had reduced QoL, although behaviour and familial cohesion were preserved, as seen in many chronic diseases. At the last assessment, in 19 patients, there were no statistically significant differences except for bodily pain and mental health, but both showed improvement. As hypothyroidism may play a very important role in QoL and educational achievement in children, there is a field for future analysis of this aspect in children who present coincidence of the two chronic diseases [17].

The above specific areas of research are summarized in Table 3.

There is a lack of prospective studies on the influence of thyroid hormone disturbances on the clinical outcome in patients with PH, especially in paediatric population. The retrospective study on thyroid function with assessment of TSH (thyroid stimulating hormone), fT3 (free triiodothyronine), fT4 (free thyroxin), and thyroid hormone replacement (THR) therapy in a large cohort of adult patients with PH showed associations with survival in specific PH subtypes [32]. Among 1756 patients enrolled in the Giessen PH Registry (Germany), in the years 1999–2013, 355 had PAH, among them 192 IPAH, 533 PH due to left heart disease, 498 PH due to lung diseases, and 370 chronic thromboembolic PH (CTEPH). The prevalence of thyroid disease was 25% overall and 28% in patients with IPAH. The prevalence of hypothyroidism and AITD in patients with PAH was 13% and 4%, respectively. In patients without THR therapy, both abnormally high and low TSH levels were associated with a worse outcome in IPAH, even after adjusting for other parameters. Furthermore, lower fT3 levels were independently associated with increased mortality in patients with PAH and CTEPH. Lastly, the absence of THR treatment was also independently associated with increased mortality in patients with IPAH. The authors presented a hypothesis of negative influence of imbalanced thyroid function on right heart function by multiple mechanisms [32]. However, the coincidence of low fT3 may be related to the severity of the disease itself, such as in low T3 syndrome (nonthyroidal illness syndrome, sick euthyroid syndrome). It has been confirmed in several studies that low T3 syndrome may be a predictor of poor prognosis in critically ill patients with, among others, respiratory failure or cardiovascular diseases. THR therapy remains controversial in these specific clinical settings [33,34,35].

In another important analysis, the authors discuss the influence of comorbidities commonly associated with PAH diagnosis on clinical outcome in adult patients, using data from the REVEAL registry [12]. Thyroid disease, diabetes, and systemic arterial hypertension are typically associated with IPAH, but may affect all PH groups. Importantly, the authors noted that patients with comorbidities are often excluded from clinical trials, therefore limiting the results. Secondly, comorbidities can mask the PAH symptoms and can lead to delay in diagnosis and, afterwards, in difficulty in evaluating the clinical outcome. Many of these diseases lead to increased pulmonary artery pressure and worsen the PAH condition. The proper management of the comorbidity may also improve the PAH outcome. The authors discuss the influence of both hypothyroidism and hyperthyroidism on increasing cardiac output and pulmonary vascular resistance, which may contribute to PH. Therefore, thyroid function tests should be not only a part of the initial evaluation at the moment of PH diagnosis but also further monitoring is needed, with the investigation occurring at least once a year or in cases of rapid PH deterioration [12].

The authors of the 2019 update on definition, classification, diagnostics, and management in paediatric PAH, on the basis of the Sixth World Symposium on PH, presented a valuable analysis of Down syndrome as an illustrative example of the role of comorbidities in paediatric PH [8]. Down syndrome is associated with cardiovascular and pulmonary morbidity and mortality in children, including PH. Recent genetic studies showed that three antiangiogenic (antivascular endothelial growth factor) genes are present on chromosome 21: encoding endostatin, RCAN-1 (regulator of calcineurin-1), and β-amyloid peptide. These findings suggest that they may lead to decreased lung vascular and alveolar growth and the further risk of PH. As the phenotype of Down-syndrome-related PH is variable, it was agreed that children with Down syndrome may be classified as PH group 3 in the absence of CHD (PH group 1 or 2, Table 1) [8]. Moreover, the increased risk of AITD and hypothyroidism in patients with Down syndrome in general, and specifically with diagnosis of PH, make future genetic studies on the possible common autoimmune background more interesting.

We need to remember that hypothyroidism may influence PH treatment and vice versa. In case of simultaneous diagnosis of hypothyroidism in a patient with a newly detected PH, there is a need for careful dosage of levothyroxine in a titrating manner. This type of initiating thyroid hormone replacement is as important as in other cardiac conditions to also avoid iatrogenic hyperthyroidism [17]. There is still an open question whether to treat subclinical hypothyroidism, with fT3 and fT4 within the normal range and an elevated TSH, with the data coming from the studies on adult patients with PH. Some authors suggest consideration of closely monitored treatment [28], especially in view of the results of the study cited above that revealed the negative influence of lack of THR therapy on mortality in patients with IPAH [32]. Another study presented high frequency of subclinical hypothyroidism: 49.1% of 53 adult patients with PAH; 84.8% of the participants were women. The mean TSH level was 4.2 mIU/mL (0.7–10) and significantly correlated with the right ventricular and diastolic dimension. The authors concluded that impaired thyroid function is most often related to right ventricular failure. Further studies are needed to prove whether THR treatment may be beneficial in such circumstances [36]. On the other hand, the possible coincidence of PH and AITD and hypothyroidism makes the necessity of cardiological examination at the moment of hypothyroidism diagnosis even the more important issue.

There is still a need for future specific paediatric studies on PH, which has been concluded by the authors of recent updated consensus statements on paediatric PH and PAH [8,37]. As the disease is rare, data from international registries are valuable and allow the analysis of large cohorts of patients. Another approach is to create centralized national services with a protocolized approach, which gives an opportunity to study the national epidemiology and outcomes in paediatric PH while limiting selection bias [11,25]. Finally, it is worth underlining that children with PH may need multidisciplinary care, including PH physicians, endocrinologists, geneticists, dietary specialists, and psychologists, due to the frequent coexistence with genetic disorders, mental delay, hormonal abnormalities, and growth impairment [7,31].

## 3. Conclusions

Despite the fact that thyroid disorders are not included in a recent PH classification, it is important to remember this possible coincidence, especially in cases of PAH in the paediatric population. Clinicians should be aware that thyroid dysfunction may be closely associated with PH prognosis, treatment outcome, and growth and development processes in children. Both diseases may also contribute to impairment of QoL and educational achievement in this group of patients. Therefore, a multidisciplinary approach is postulated in children with PH. Future specific studies of mutual influence of AITD and hypothyroidism and PH in paediatric populations are needed.

## Figures and Tables

**Table 1:** Prospective studies on pulmonary hypertension in children.

Year of Publication [Reference]	Authors	Registry, Country, Years of Study	n	Age of Patients	IPAH n (%)	Hypothyroidism n (%)	Down Syndrome n (%)	Other Genetic Anomalies n (%)
2009 (PAH study) ^a^ [16]	van Loon, R.L.E. et al.	Netherlands, 1993–2007	63	Median at enrolment 5.8 (0.1–17.4)	29 (46)	NG	13 (21)	14 (22.2) 2: Noonan syndrome, 7: undefined
2010 [17]	Fraisse, A. et al.	France, May 2005–June 2006	50	Mean at enrolment 8.9 ± 5.4	30 (60)	NG	NG	NG
2012 [18]	Berger, R.M. et al.	TOPP, 19 countries, 2008–2010	362	Median at diagnosis 7 (3–12)	IPAH/FPAH 317 (88)	1- Thyroid disease	Chromosomal anomalies, mainly DS 47 (13%)	NG
2012 [19]	Barst, R.J. et al.	REVEAL, US, 2006–2010	216	Median at diagnosis 7, at enrolment 15	IPAH/ FPAH 122 (56.5)	NG	NG	NG
2020 (PAH study) ^b^ [7]	Kwiatkowska, J. et al.	Poland, March 2018–September 2018	80	Median at diagnosis 5.1 (2.1–8.1), at enrolment 10.4 (7.9–15.2)	25 (31.25)	19 (23.8)	24 (30)	9 (11.3) 2: Noonan syndrome

N: number of patients; IPAH: idiopathic pulmonary arterial hypertension; FPAH: familial pulmonary arterial hypertension; NG: data not given; DS: Down syndrome; US: United States; TOPP: The Tracking Outcomes and Practice in Pediatric Pulmonary Hypertension; REVEAL: The Registry to Evaluate Early and Long-Term PAH Disease Management. ^a^ Network for Diagnosis and Treatment of Pediatric PAH, ^b^ Polish Registry of Pulmonary Hypertension.

**Table 2:** Retrospective analyses on pulmonary hypertension in children.

Year of Publication [Reference]	Authors	Registry- Country, Years of Study	n	Age of Patients	IPAH n (%)	Hypothyroidism n (%)	Down Syndrome n (%)	Other Genetic Anomalies n (%)
2010 (IPAH study) [20]	Moledina, S. et al.	UKSPHC ^a^, UK, 2001–2008	64	Median at diagnosis 4.3 (1.5–8.9), at enrolment 6.5 (2.6–13)	NA	NG	3	1: Noonan syndrome 1: Triple X syndrome
2011 [21]	van Loon, R.L.E. et al.	Netherlands, 1991–2005	3263, 603-persistent PAH, 2660- transient PAH	Median at diagnosis 2.2 (0.4–6.7) *	Progressive PAH 154 (25.5) **	NG	27 (18)*	33 (21.4) 2: Noonan syndrome, 17: undefined *
2014 [22]	Marin, M.D.C. et al.	Spain ^b^, 2009–2012	225	Mean 4.3 ± 4.9	IPAH/ FPAH 31	32 (14.3)	36 (17)	50 (22.2)
2016 [15]	Ploegstra, M.J. et al.	4 registries ^c^	601	Median 9.1 (5.1–13.6)	IPAH/ FPAH 337 (56)	NG	65 (11)	NG
2017 (PAH study) [23]	Li, L. et al.	US, 2010–2013	695	4.8–8.1	NG	19 (2.7): Thyroid diseases	87 (12.5)	102 (14.7) 52: undefined
2022 [24]	Abman, S.H. et al.	PPHNet ^d^, US, 2014–2020	1475	Mean 2.9 ± 4.7	663 (44.9)	65 (4): Thyroid replacement therapy	158 (10.7)	91 (6.2) 7: Noonan syndrome
2022 [25]	Constantine, A. et al.	UK ^e^, 2001–2021	1101	Median 2.6 (0.8–8.2)	PAH 529 (48%), of these IPAH 22.3%	NG	176 (16)	194 (17.6)

N: number of patients; IPAH: idiopathic pulmonary arterial hypertension; FPAH: familial pulmonary arterial hypertension; NA: not applicable; NG: data not given; UK: United Kingdom; US: United States; * data regarding progressive PAH; ** in relation to persistent PAH. ^a^ United Kingdom Service for Pulmonary Hypertension in Children (UKSPHC); ^b^ Spanish Registry for Pediatric Pulmonary Hypertension; ^c^ TOPP: The Tracking Outcomes and Practice in Pediatric Pulmonary Hypertension; REVEAL: The Registry to Evaluate Early and Long-Term PAH Disease Management, Dutch (Network for Diagnosis and Treatment of Pediatric PAH) and French national registries; ^d^ Pediatric Pulmonary Hypertension Network (PPHNet); ^e^ United Kingdom National Pediatric Pulmonary Hypertension Service.

**Table 3:** Summary of specific areas of research regarding possible association of thyroid diseases and PH: genetic background, growth restriction, and QoL in children with PH.

Area of Research	Year of Publication [Reference]	Authors	Study Group and Main Results	Main Conclusions
Possible common genetic background of both thyroid diseases and PH	2005 [29] short report	Roberts, K.E. et al.	75 children and 66 adults with IPAH. 5 novel *BMPR2* mutations in 4 adults (6%) and in 1 child (1%), all had thyroid disease (100%), which was present in 24% of adults and 5% of children without *BMPR2* mutations.	Possible association of thyroid disease and *BMPR2* mutations in patients with IPAH.
	2022 [30]	Cruz-Utrilla, A. et al.	98 children with PH. Pathogenic or likely pathogenic variants in 44 patients (44.9%), the most frequently affected gene: *BMPR2* (10 cases, 64.7%). 28.6% of the study cohort was reclassified regarding PH group.	Possible change of PH clinical classification with prognostic implications after genetic testing.
Growth restriction in children with PH	2020 [7]	Kwiatkowska, J. et al.	80 children with PAH. Severe growth retardation (height below third percentile for age) in 30% of patients with CHD-PAH and 16% with IPAH.	Need for cooperation between PH physicians, endocrinologists and dietary specialists.
	2010 [20]	Moledina, S. et al.	64 children with IPAH. Mean weight z-score: −0.66 SDS, height z-score: −0.71 SDS.	Association of low weight z-score with increased mortality in children treated for IPAH.
	2010 [17]	Fraisse, A. et al.	50 children with PAH. Low weight in 12 patients (24%) and height in 8 (16%).	Significant part of children with PAH presented growth retardation.
	2022 [25]	Constantine, A. et al.	1101 children with PH. Median weight z-score: −1.4 SDS (−2.7 to 0.3), height z-score: −1.1 SDS (−2.1 to 0.1).	Growth restriction was greater in children with PH due to lung disease vs. PAH.
	2016 [15]	Ploegstra, M.J. et al.	601 children with PAH. Median height for age z-score: −0.81 SDS (−0.93 to −0.69) and BMI for age z-score: −0.12 SDS (−0.25 to −0.01).	Improvement of PAH clinical course was associated with catch-up growth. Height for age could be an easy to implement parameter to assess the PAH treatment outcome.
QoL in children with PH	2010 [17]	Fraisse, A. et al.	81% of 40 patients with PAH over 3 years old were attending regular school. The majority of children with PAH over 5 years old had reduced QoL. At the 2-year assessment, the scores for bodily pain and mental health were reduced but both showed improvement.	Combined PAH-specific therapies may lead to relative stability in QoL.

PH: pulmonary hypertension; QoL: quality of life; PAH: pulmonary arterial hypertension; IPAH: Idiopathic PAH; *BMPR2*: bone morphogenetic protein receptor type II gene; CHD-PAH: PAH associated with congenital heart disease; SDS: standard deviation score; BMI: body mass index.

## Data Availability

No new data were created or analysed in this study. Data sharing is not applicable to this article.
